# Asiatic Cholera

**Published:** 1866-08

**Authors:** 


ASIATIC CHOLERA. By F. A. BURRALL, M.D. WM. WOOD & CO., 61 Walker Street, New York. 1866.

This is a duodecimo of 155 pages, neatly printed, and contains a summary of the prevalent doctrines relating to the communicability, symptoms, and treatment of cholera. We have failed to find in it any substantial additions to the stock of knowledge previously accessible to the profession.

